# How can Quality of Life be Achieved in a Sustainable Way? Perceptions of Swiss Rural Inhabitants

**DOI:** 10.1007/s43621-022-00114-6

**Published:** 2022-12-01

**Authors:** Thea Xenia Wiesli, Thomas Hammer

**Affiliations:** Centre for Development and Environment CDE, University of Bern, Mittelstrasse 43, 3012 Bern, Switzerland

**Keywords:** Quality of life, Well-being, Sustainability, Rural areas, Regional development, Europe

## Abstract

Rural regions in Europe are often structurally weaker than urban areas and are subject to strong socio-economic development. At the same time, they offer opportunities for a high quality of life and sustainability. The key question of this article is how quality of life in high-income countries can be achieved more sustainably. Little is known about the perception of the rural population itself on the reconciling of high quality of life with sustainability. Thus, based on a concept of sustainable quality of life, qualitative interviews with 90 rural residents were conducted to ask them which factors benefit sustainable quality of life. In the perception of the interview participants, a change in attitudes and values would be a starting point for shaping many other areas to enhance sustainable quality of life; social and legal norms should provide reference points for individuals and economic actors; infrastructure should support individuals in their ecological behaviour; and the economy should serve the common good. We derive four strands of recommendations for decision-makers from these results: the enhancement of education on applicable environmental behaviours, equal access to renewable energies and local productions and services.

## Introduction

Although basic material needs are a prerequisite for quality of life (QoL) [1],^1^ it can be assumed that consumption only has a limited influence on QoL [4, 5]. Instead, excessive consumption of natural resources can cause environmental and social problems and thus decrease QoL [6–8]. A sustainable quality of life (SQoL) would mean QoL would not only be guaranteed for some individuals or societies but for all present and future generations globally [9]. Several studies indicate, that most countries globally do not yet manage to achieve a high QoL which is sustainable at the same time [4, 5]. Nevertheless, this does not preclude the possibility of wealthy nations reducing the negative global impacts of over-consumption without impairing QoL [4, 5, 10–12]. A key question, however, is how QoL can be achieved in a sustainable way [13].

The initial conditions for QoL and its sustainability (e.g. possibilities for environmentally friendly behaviour) vary by region and by the level of urbanization [13, 14]. The different infrastructures, services, and lifestyles of urban and rural area types require differentiation of public policies in relation to the living environment. In this article, we focus on rural areas in Europe.

A major trend towards urbanisation occurred during the twentieth century and Europe's rural regions have undergone major structural changes in recent decades [15, 16]. In many cases, these structural changes include socio-demographic changes, such as the ageing of the population; socio-economic changes, such as a decline of the employed individuals in agriculture; and cultural changes, caused by globalisation and urbanisation [17]. The challenges regarding QoL are particularly prominent in the service sector and the provision of infrastructure: in job security, care and health services and public transport [18–26]. On the other hand, rural regions also offer a particularly good basis for a high QoL due to good access to natural and cultural landscapes, the possibility of acquiring residential property and a sense of belonging to the local community and security [18, 26–30]. A larger extent of quantitative studies has been examined on (sustainable) QoL depending on the degree of urbanisation and rurality [13] and suggests that inhabitants of rural or intermediate rural areas in Europe [18, 31, 32] and in the USA [33] have a significantly higher life satisfaction than inhabitants of urban areas. In some cases, as in rural Western Europe, residents benefit indirectly from the advantages of urban regions without suffering from the negative effects of cities [32]. In addition, rural areas offer a high potential for the implementation of sustainable regional economic approaches and value creation through regional and seasonal products. Furthermore, rural areas can encourage a lifestyle in which cultural heritage, identity, originality and self-determination, as well as “community-based and charitable action”, play a more important role than consumption [17].

While so far research provides valuable insights into the challenges and differences in the QoL in rural areas, often from a quantitative perspective, to our knowledge rural inhabitants’ perception on linking QoL with sustainability is hardly known. In order to support sustainable development, it is essential that decision-makers are aware of public perception, as this means they can develop strategies and initiate measures that are viable and supported by society [34]. Hence, the goal of this paper is to ascertain rural inhabitants’ perceptions of the factors beneficial for SQoL, and derive recommendations for decision-makers of rural areas from these findings.

The study draws on qualitative interviews with Swiss rural inhabitants. Switzerland, scores above average in terms of QoL and resource consumption [1] and, thus, Swiss rural regions serve in this article as a suitable example of the dilemma between high QoL and sustainability in high-income countries. As in other European countries, major changes in urbanisation have taken place in Switzerland in recent decades. At the same time, Switzerland offers an example of a relatively dense population (app. 215 people per square kilometre) and a high proportion of rural settlement areas, as a large part of the migration from mountain regions has ended up in the relatively well-accessible villages and agglomerations near the cities [35].

In the following sections, our concept of “Sustainable Quality of Life” will be summarised, the qualitative approach explained and the benefits for SQoL in the perception of the rural inhabitants will be presented. Accordingly, we provide recommendations for local decision-makers to enhance SQoL in rural areas.

### Sustainable quality of life

The concept “Sustainable Quality of Life” (SQoL) in Wiesli et al. [9] served as the theoretical basis for this article. Following, the United Nations’ (UN) understanding of sustainable development [36], SQoL is defined concretely as high QoL in a healthy environment, without overuse of natural resources, for present and future generations globally. Accordingly, social, equality and ecological demands are linked to high QoL. QoL is thus not only seen from the perspective of individuals but from an intragenerational and intergenerational perspective [9]. Our literature analysis and the subsequent comparison of the results with rural inhabitants’ perception, by means of qualitative interviews in Wiesli et al. [9], showed that SQoL must be considered a multidimensional concept. The concept consists of nine components (see Table 1): “Social relations and equality, participation, identification and collective emotions, nature and landscape, education and knowledge, leisure and recreation, living, health and safety, mobility, and income and work” [9]. The distinguishing feature of the concept is that each component integrates social, economic and ecologically sustainable development into the dimensions of QoL.

**Table 1:** Components of the sustainable quality of life concept [9]

Component	Target
Social relations and equality	The greatest possible freedom and equal chances. Intra- and intergenerational justice. Opportunities for social relations. No discrimination based on gender, ethnicity, religion, species or other affiliation. Recognition of potentially excluded groups as a basis for (environmental) justice.
Nature and landscape	High quality nature and landscape for all present and future generations
Education and knowledge	A good general and specific education and knowledge. Education on the environment and sustainability. The ability to absorb and process information, think critically and use one’s personal knowledge.
Living	Appropriate, environmentally friendly and resource-efficient living conditions that are not impaired by environmental pollution.
Health and safety	A long and healthy life without fear, the danger of conflicts or negative environmental and climatic influences.
Participation, identification and collective emotions	Freedom of choice, the right to have a say and effective participation in social processes. Identification with one’s social environment and home area. A positive collective mood and common managements.
Mobility	Environmentally friendly and resource-efficient mobility for everyone, including efficient and frequent access to cities.
Leisure and recreation	Leisure activities, recreation and cultural activities that are as environmentally friendly as possible and compatible with the conservation of renewable natural resources.
Income and employment	Employment within the framework of a resource-efficient and environmentally friendly economy. Employment that is freely chosen and meaningful and provides sufficient income and a good work-life balance.

## Materials and methods

### Study regions

The 90 interviews were conducted in municipalities in the four large Swiss regions Gantrisch, Freiamt, Entlebuch and Aargauer Jura [9]. All four areas are in the German-speaking part of Switzerland and contain several types of rural municipalities with typical characteristics for a large proportion of Switzerland’s rural areas, such as the infrastructure, middle or larger population density (e.g. 168 inhabitants per square kilometre in the Aargauer Jura) and landscape characteristics including lowland and mountain areas [23]. We planned to include a diversity of perspectives and, thus, to conduct at least 20 interviews per region. We selected the municipalities according to the typology of municipalities published by the Swiss Federal Statistical Office [37]. The majority of the individuals in the sample are residents of peri-urban municipalities or rural but centrally located municipalities. These types of peri-urban municipalities are the most common municipality type in rural areas [38].

In these rural areas, major structural change has taken place as in other OECD-countries [39]. In recent years, significantly more individuals started to work in the service sector and significantly fewer individuals work in agriculture [38]. Due to the decline of basic service supply, the distances required for reaching basic services have increased. In peri-urban rural areas, the geographical separation of work and living space has led to heavy commuter flows to the cities, which constitute a challenge for mobility infrastructures [38]. Furthermore, higher educational and occupational requirements have arisen in rural regions of Switzerland [39]. In contrast to several European countries, the rural population of Switzerland (especially in peri-urban municipalities) has increased by 7% since 2000 [38]. In peripheral rural areas, however, migration to other areas and demographic ageing are common [38]. Ecologically valuable areas in rural Switzerland are threatened by ground sealing, fragmentation, pollution and intensification of land use practices [38]. All of these challenges can be seen as comparable to similar other European countries. Our investigations in these areas, thus, can give hints for the enhancement of SQoL in comparable European countries.

### Procedure of data collection

A semi-structured interview guide with open and narration-generating questions was developed for the 90 interviews. The interview guide can be consulted in the supplementary materials of Wiesli et al. [21]. Before we conducted the actual interviews from June to August 2019, we subjected the interview guide to several test interviews and adjusted it accordingly. The interview questions were posed according to the content of the participants’ discussion as opposed to following a strict question chronology. In order to discuss the term “sustainability” based on a common understanding, the definition of sustainability used in the concept of SQoL was explained: “We understand it to mean that all people in the world have a good life now and in the future without harming the environment” [21]. The interviews lasted on average 40 min. With the participant’s consent, a voice recording was made of the interview.

The aim of the interviews for this article was to identify the main factors perceived as beneficial for SQoL by the rural inhabitants. For this purpose, the interview participants were asked to describe beneficial factors to the combination of high QoL with sustainability (see results in Sect. 3).

### Sampling and participants

The sampling method was conducted according to the theoretical sampling by Glaser and Strauss [40]. The participants were randomly selected by asking in the villages to be part of an interview and avoided snowball sampling to minimize sociodemographic biases [21]. We recruited participants in public places, such as in front of stores, schools, train stations, in the field or in the village square in consideration of the sampling targets. Before the interview, we informed the participants in detail about the recording, the anonymous and coded use of the interview and about the publication of their statements and asked them for their consent to the use of their statements. Furthermore, we asked the interviewed participants if they were interested in receiving the results of the studies as a report. Interested participants were sent a short report after the analysis and evaluation of the interviews, which was written especially for them. The addresses, we collected for this purpose were encrypted and subsequently deleted.

The demographical characteristics, namely sex, age, education, occupation and place of residence classified according to municipality typology, were recorded and checked during the whole field research in order to obtain a balanced sample. We, thus, adjusted the sampling to the sociodemographic situation of the villages and included participants with diverse backgrounds as much as possible. Towards the end of the field research, demographical groups who have not been involved in the sample were targeted and included in the sample.

The proportion of men and women in the sample was almost equal; 44 men, 46 females (see Table 7 in the Appendix). Three different age groups are included in the sample; young (starting from 16 years), middle (starting from 29 years) and elderly (starting from 60 years). The middle group, the 29- to 59-year-olds, is the largest group in the sample. The majority of people in the sample held a secondary school diploma. This diploma corresponds to basic vocational training or an upper secondary school diploma (level 3) and entitles the holder to study at higher educational institutions such as universities. Most participants are employed in the service sector. In other rural regions of Switzerland, women are also slightly more than men, 29- to 59-year-olds also constitute the largest age group and the majority of people in Switzerland work in the service sector [41]. Most people in Swiss rural areas have a secondary school degree [42]. Hence, regarding these socio-demographic characteristics, we assume that the sample corresponds to the Swiss rural average. In addition, many statements in the interviews were similar after less than half of the interviews and we were able to detect saturation of the content. We, therefore, assume that the statements apply to many individuals in Swiss and comparable European rural regions. However, based on our data from the 90 interviews and the qualitative methods used, the transferability of the socio-demographic characteristics and the messages in the interviews to other contexts is limited.

### Content analysis

The recorded interviews were transcribed according to the semantic content transcription method [43, 44]. Thus, the content in Swiss German was transcribed literally and not in summary. The qualitative content analysis was carried out according to Mayring's [45] method. Using this method, the categories were initially generated deductively on the basis of theory, and, after 90 interviews had been conducted, additionally supplemented inductively from the text content. The deductive codes were derived from the SQoL concept and its components. A coding guide contained the coding rules and anchor examples, which specified what the content of the interviews needed to contain in order to fall into the respective categories (see codebook in Wiesli et al. 2021, supplementary material).

In order to evaluate the perception of rural inhabitants regarding factors benefiting SQoL, the two categories “benefits” and “obstacles” were deductively formulated. Corresponding statements by the participants were assigned to these two categories and inductive sub-codes were continuously formulated.

The new codes were invented during the first 40% of the interviews [21]. After this, no new benefits or obstacles that would have led to new sub-codes were identified in the analysed transcripts. The intercoder reliability between the two researchers was tested and newly created sub-codes were afterwards communicated among the researchers using the exact descriptions in the coding guide and discussed in order to avoid ambiguities.

During the final evaluation, it was found that the codes with the obstacles implicitly always corresponded to benefits to SQoL. Therefore, the sub-codes of the obstacles were classified into the category of benefits. Finally, in order to arrive at a superordinate structure, as suggested in Mayring's approach [45], categories into which all benefits to SQoL could be classified were searched inductively and interpretatively. In this way, four categories of benefits to SQoL were created.

## Results

The four categories of benefits to SQoL were named “attitudes and values”, “social and legal norms”, “physical infrastructure” and “economic structures” (see Table 2). These four categories are introduced in the following. Afterwards, the results of these categories and their mapping into the nine components of the concept SQoL are presented.

**Table 2:** The four categories of benefits to SQoL based on the perception of the interview participants

Attitudes and values	Social and legal norms	Physical infrastructure	Economic structures
Values are represented in many and varied ways and determine what is important or less important. Attitudes constitute values and are manifested as evaluations of states, objects and behaviours.	Social norms are rules that most members of society use to guide their behaviour. Legal norms are enshrined in law and can also be enforced independently of consent.	Physical infrastructure includes facilities, equipment and buildings that are available for communal use and co-determine people's behaviour.	Economic structures include facilities, actions and systems that serve the organised satisfaction of human needs with goods and services.

The first thing that stood out during the coding process was, that a larger part of the benefits of SQoL was related to attitudes and values. For example, some participants mentioned personal responsibility or specific political perceptions as beneficial factors for SQoL. Hence, the category “attitudes and values” was created. In accordance with Schwartz [46], values are understood as something that individuals represent in many different ways and that determine what is important or less important to individuals in their lives. Attitudes are determined by values and take the form of an evaluation of “states”, “objects and behaviour” ([46], p. 16).

It was also discovered that some of the aforementioned benefits to SQoL relate to social and legal norms. According to a large proportion of the interview participants, social and legal standards are necessary to achieve SQoL. Based on Schwartz [46], social norms are understood as rules that most members of society use to guide their behaviour. Values determine whether social norms are accepted or not [46]. Social norms are not necessarily subject to official laws and are not necessarily enforced or sanctioned. In the perception of the interviewed participants, norms contributing to SQoL should be socially rooted in society. In contrast, legal norms are defined as norms that are enforced by authorities and can be imposed without the consent of individuals. The majority of participants primarily called for legal standards that would govern how natural resources could be used.

In addition, the interview participants named some benefits to SQoL that relate to the existence of appropriate physical infrastructure. Thus, the category “physical infrastructure” was formulated. “Physical infrastructure” is defined as equipment and buildings that are available to the community and support its activities. Beneficial factors of this kind refer in particular to participants' environment and prevailing circumstances. According to the participants, these should be improved in terms of SQoL in order to enable individuals to behave accordingly.

Further benefits for SQoL that were mentioned, such as “sustainable tourism”, were assigned to the category “economic structures”. The category “economic structures” is defined as the totality of all facilities, actions and systems that serve the organised satisfaction of human needs through goods and services. Beneficial factors of this kind primarily concern various areas of the economy that, in the perception of the participants, should be aligned with the interests of the broader population, for example by ensuring fair distribution.

### Benefits to SQoL in the category of attitudes and values, based on the perception of the participants

Three benefits for SQoL can be classified under the category “attitudes and values”: attitudes towards sufficiency, personal responsibility and a common political perception that is compatible with SQoL (see Table 3). Two-thirds of the participants saw an attitude of sufficiency as beneficial to SQoL. From their point of view, individuals with this attitude are satisfied with a certain contingent of goods and services and do not strive for further consumption. Personal fulfilment should furthermore be less strongly connected to material values. On the one hand, this would lead to higher satisfaction, and on the other hand, lower consumption would reduce resource overuse and inequality. An interview participant explained:

*“In principle, every luxury is harmful to the environment. If everyone lived more simply, we would have far fewer problems. Then it would be enough even for those who have less.”* (IP 55, >60 years, male, retired, secondary school degree, peripheral municipality)

**Table 3:** Benefits to SQoL in the category of attitudes and values, based on the perception of participants, and the corresponding components from the SQoL concept

Benefits to SQoL in the category of attitudes and values	Mentioned by …% of 90 participants	Mentioned by … people out of 90 participants	Concerns component … in the SQoL concept^a^
The majority of individuals represent an attitude of sufficiency.	65.5	59	Concerns several components
The majority of individuals take personal responsibility.	62.2	56	Concerns several components
Society and its parties have a common political stance, which is oriented towards SQoL.	20	18	Concerns all components

^a^A maximum of four components is listed in the tables.

According to these respondents, and with regard to the nine aspects that constitute the concept of SQoL (see Table 1), an attitude of sufficiency could benefit all areas of life in which lower resource use contributes to SQoL. This beneficial factor could thus contribute to several aspects of SQoL, such as mobility, living and leisure and recreation. Overall, sufficiency would mainly support the component of *social relations and equality*. This is because, according to the respondents, frugality reduces the overexploitation of resources, which means that more goods and services are available and can be distributed more evenly.

Furthermore, two-thirds of the participants believe that an attitude of personal responsibility would be conducive to SQoL. They argued that individuals should frequently consciously reflect on their own behaviour, think about the consequences of their own actions and thus behave in a more solidary and responsible manner. This behaviour is related to different areas, such as resource overuse, climate change, empathy and helpfulness towards other people.

*“I believe that everyone can start with themselves. This was also a reason why we came here, because of the heating, so that we don't need the car and so that the children can play outside. We didn't have a television for a long time either so that the children could experience something other than just consumption.”* (IP 50, 30–59 years, female, service, tertiary degree, peri-urban municipality)

This benefit to SQoL, as stated by the participants, relates to several components. Responsible behaviour, which leads to more ecological behaviour, mainly affects components such as *mobility*, *living, work and income, nature and landscape, leisure and recreation* and *education and knowledge.* Responsible behaviour, which leads to empathy and helpfulness, can contribute to the components of *social relations and equality* and *participation, identification and collective emotions.*

About one-quarter of the participants see specific political perceptions as conducive to SQoL. The participants mainly referred to a progressive political perception in line with sustainable development and interest in common QoL. Some of these respondents also believe that a consensus among villages on political matters could contribute to freedom of expression and participation on the part of the village population. This could strengthen social relations between people. Some individuals among this participant group described themselves as politically left or green, and see politically conservative, right or populist attitudes as inhibiting the progress of processes and developments in society.

*“For example, when it comes to gays and lesbians or foreigners. We are still too right-wing in Entlebuch.”* (IP 75, 30–59 years, male, service, secondary degree, rural central municipality)

Political attitudes compatible with SQoL could contribute to all SQoL components. Above all, the consensus among individuals could strengthen freedom of expression, common emotions and a sense of belonging, and thus could contribute to the component *participation, identification and collective emotions*.

### Benefits to SQoL in the category of social and legal norms, based on the perception of participants

Six benefits to SQoL were assigned to the category of “social and legal norms” (see Table 4). A significant proportion of participants saw legal norms, such as laws, sanctions and price regulations, as important for achieving SQoL. Other participants opined that civic engagement and changed habits should be established as social norms in order to direct society towards SQoL. Further beneficial factors mentioned by participants concerned nature conservation, sustainability and environmental education and changes in agricultural policy.

**Table 4:** Benefits to SQoL in the category of social and legal norms, based on the perception of participants, and the corresponding components from the SQoL concept

Benefits to SQoL in the category of social and legal norms	Mentioned by …% of the 90 participants	Mentioned by … people out of the 90 participants	Concerns component … in the SQoL concept
Laws, sanctions and price regulations supporting SQoL exist.	75.5	68	Concerns several components
Sustainability and environmental education is aimed at specific target groups.	45.5	41	Concerns several components
Individuals get involved in civil society.	40	36	Concerns several components
Individuals change their habits	23.3	21	Concerns several components
Nature and species are strictly and widely protected.	20	18	Nature and landscape Leisure and recreation Education and knowledge
Agricultural policy is based on the quality of production methods.	5.6	5	Income and employment Nature and landscape

The majority of participants (75.5%) saw legal standards and thus legal regulations and sanctions as necessary to achieve SQoL. More than half of these participants were in favour of legal regulations to reduce plastic waste. From their point of view, reducing household waste is impeded by food packaging from wholesalers. In this context, people in the youngest age group (16–29 years) mentioned no-waste stores, presently mainly found in cities, and indicated that they would like to see the opening of such stores in their villages. People in the oldest age group (> 60 years) stated that they would like to refill more products, as they used to do in shops in the past. A third of these participants also suggested legal requirements for spatial planning, restrictions on long-distance animal transport, higher prices for petrol, the declaration of carbon emissions on product packaging and greater transparency regarding the origin of products.

*“Petrol needs to be much more expensive, even if it would hurt me. We also drive a little bus, but petrol needs to be more expensive [...]. Wholesalers would have to be obliged to declare the facts, and not write “market-fresh grapes” even though they are from Israel or someplace like that. They should be forced to inform consumers better.”* (IP 44, 30–59 years, female, service, secondary school degree, peri-urban municipality)

Laws, sanctions and price regulations mentioned by participants concern several components of SQoL, in particular *mobility, living, work and income, nature and landscape* and *education and knowledge.*

Similarly, about half of the participants are of the perception that sustainability and environmental education should be part of the educational curriculum and this way established as a social norm. From the point of view of these respondents, some people are not sufficiently aware of intergenerational and global equality and had insufficient knowledge about the connections between their own behaviour and global impacts. The respondents argued that this was leading to unconscious actions, in particular overconsumption, that leads to the exploitation of individuals in other countries. They further stated that even target groups that were not explicitly interested in sustainability issues would need to be reached, for example through extracurricular education. Some of these participants also claimed that they often did not know how to act more sustainably in everyday life. School and extracurricular sustainability and environmental education should therefore include specific and practical tips.

*“In my case, there is sometimes such a prevalence of pseudo-knowledge. This scares me a little. [...] There should be basic knowledge. Because, in such complex environmental issues, there would still be so much more to consider, for example, technical knowledge.”* (IP 26, >60 years, male, service, secondary school degree, peri-urban municipality)

Sustainability and environmental education essentially concern the component of *education and knowledge*. According to the interview participants, sustainability and environmental education are accompanied by a greater amount of ecological behaviour, which affects other components of SQoL (e.g. mobility). The participants also wanted all relevant target groups to be exposed to sustainability and environmental education. This demand for equality contributes to the component of *social relations and equality*.

Almost half of the participants perceive activities that increase public welfare, such as civic engagement, as conducive to SQoL. They believe it strengthened the cohesion and development of society when individuals were regularly active in organisations and associations on a voluntary basis for no material gain. These participants claimed that this was beneficial to a culture of loyalty and could lead to social and ecological developments in society.

*“I do a lot for the handicapped and I always get the idea that a lot of things are free. I don't always have to get something out of it. I don't always have to earn money. That's sustainable for me although I don't know if this is the correct meaning of sustainability.”* (IP 57, >60 years, male, retired, tertiary degree, peri-urban municipality)

With regards to the concept of SQoL, various components are indirectly or directly affected by this factor, depending on the type of activity. However, according to the participants, the resultant relationships and public welfare would primarily affect the components of *participation, identity and collective emotions* and *social relations and equality.*

A quarter of the participants see a change in personal habits as a prerequisite for SQoL. In their view, people’s everyday behaviours and actions should change in a way that rendered them compatible with SQoL. The participants explained that they sometimes behaved unsustainably out of habit, although they were aware of the negative consequences. Such behaviours include shopping habits, car driving and dietary habits.

*“You can start small and change habits. Maybe you ensure that you don’t have any leftovers so that you don't have to throw anything away.”* (IP 29, >60 years, female, services, tertiary degree, peri-urban municipality)

This beneficial factor can be applied to different components of SQoL because, according to the participants, changing habits should also change behaviour in terms of SQoL. These behaviours could, for example, lead to more sustainable *mobility* and also contribute to the components *living, work and income* and *leisure and recreation,* based on the participants’ statements.

A quarter of the participants see nature conservation as important for SQoL. They referred both to strictly protected areas and to places for leisure activities. They described low-nutrient meadows and traditional high-trunked tree species as having significant effects on landscapes and on positive emotions. Some of these participants are also convinced that nature conservation goes hand in hand with the communication of values that are beneficial to SQoL. For example, they preserve habitats for insects, reptiles and other small animals in their gardens, or save frogs from being run over by cars. According to these participants, such activities also serves, besides nature, an educational purpose for their children:

*“I think that children or the younger generations notice when you give them this [nature awareness]. This should be encouraged (laughs), so that society will eventually move away from mass commodities.”* (IP 35, female, service, 30–59 years, secondary school degree, rural central municipality)

The protection of nature affects the component *nature and landscape*. Similarly, based on the statements made by the participants, the components *education and knowledge* and *leisure and recreation* are supported because the participants regarded nature as a place where *education and knowledge* and value teaching, as well as leisure, could take place.

Five out of the 13 farmers among the interview participants are in favour of changes in Swiss agricultural policy. In their perception, instead of the area of a farm, the added value and quality of the production method should be the criterion for state financial support. In this way, smallholder farmers who farm organically would receive more support. These farmers see the current agricultural policy as un-ecological and as a mental burden that reduces their QoL, due to financial insecurities.

*“That you do not trip them up unnecessarily [...]. And that all farms are treated equally, no matter how many hectares they have. That everyone has the same rights.”* (IP 89, male, 16–29 years, forestry and agriculture, tertiary degree, rural central municipality)

The call for more ecological farm management concerns the component *nature and landscape* of the concept SQoL. Furthermore, the component *work and income* is affected, as the participants hoped that a change in agricultural policy would improve income.

### Benefits to SQoL in the category of economic structures, based on the perception of participants

Five benefits to SQoL are classified as “economic structures”: the availability of seasonal and locally produced products; the availability of local gastronomy, cultural attractions and shopping facilities; an economy based on the interests of the general population; an increase in added value; and sustainable tourism (see Table 5).

**Table 5:** Benefits to SQoL in the category of economic structures, based on the perception of participants, and the corresponding components from the SQoL concept

Benefits to SQoL in the category of economic structures	Mentioned by …% of the 90 participants	Mentioned by … people out of the 90 participants	Concerns component … in the SQoL concept
Regional, seasonal and locally produced products are available locally.	68.8	62	Mobility Nature and landscape
Restaurants, cultural attractions and shopping facilities are available locally.	28.8	26	Participation, identification and collective emotions Income and employment Leisure and recreation Social relations and equality Mobility
The economy is fairly distributed in line with the interests of the population at large.	24.4	22	Income and employment Leisure and recreation Social relations and equality
The added value of the products is higher.	18.8	17	Income and employment Social relations and equality
Tourism is sustainable and stronger.	15.5	14	Income and employment Leisure and recreation Nature and landscape

Around three-quarters of the participants sees regional products and their direct marketing as a benefit to SQoL. They associate local foods with better quality, enjoyment, higher animal welfare, sustainable agriculture and the region’s heritage, culture and tradition. However, in the participants’ perception, regional products are often hard to purchase even though they were produced in the region. As a result, the participants mainly use their cars when food shopping, although they see car-driving as harmful to the environment; the alternative was not buying local products. For this reason, 20% of these participants suggest direct marketing at weekly markets in their respective villages.

*“This should actually be brought together somewhere. You don’t go shopping in such a way that you buy potatoes from one person and then drive ten minutes so that you can buy meat from the next. So direct marketing is good, but somewhere there would have to be a place where you bring it all together.”* (IP 49, 30–59 years, service, tertiary degree, peri-urban municipality)

This proposal can be ascribed to several of the nine components of SQoL, as it could support different areas of life. The benefit to SQoL of regional products and direct marketing can contribute to *mobility,* as, according to the participants, it creates shorter distances for produce to travel. The component *nature and landscape* can also be supported, as regional products would, in the participants’ view, lead to more sustainable agriculture. By promoting local traditions and heritage culture, the component *participation, identity and collective emotions* could also be supported.

In the perception of about one-third of the participants (mostly in the 16–29 and 30–59 age groups), local shops, cultural attractions, services and restaurants benefit SQoL. In their view, these services are increasingly disappearing in rural areas. However, they see it as important that such services were maintained. In their perception, these services would help to maintain jobs and cultural and social village life, and prevent migration of the population to cities.

*“What scares me is when a shop or restaurant closes and nothing happens to it for months or a year. [...] Then village life is not about sitting together once a week in the evening and drinking a beer, but instead, people are simply at home. [...] I think community suffers like this.”* (IP 26, >60 years, male, service, secondary school degree, peri-urban municipality)

Based on the participants’ statements, local restaurants, cultural attractions and shopping facilities could thus contribute to the component *income and employment* of SQoL. Restaurants and cultural attractions are also places for leisure and social life, according to the participants. They could therefore contribute to the component *leisure and recreation* and *social relations and equality*. As restaurants, cultural attractions and shopping should, according to the participants, stay as local as possible, the component *mobility* could also be supported by reduced driving.

A third of the participants believe that a change in the overall economic structures is necessary to achieve SQoL. They see large companies as hegemonic players in our society and other members of society as powerless against them. These participants, therefore, see measures directed at making such players more accountable for appropriate tax payments and carbon emission compensation as an opportunity to support SQoL. Some criticised the one-sided focus and wage conditions of the current economic system, which is focused on growth. They also criticised the resultant increase in goods and consumption as well as in workload, pressure to perform and unequal distribution of wealth and income. They believe that economic structures should instead be geared towards the interests of the broader population.

*“I don’t think we are on such a good path. Especially for the future. And yes, everything is so unfairly distributed. Many have a lot and many have very little. I think we should definitely change something [...]”* (IP 66, 16–29 years, female, basic school degree, in education, rural central municipality)

Based on the participants’ statements, this benefit to SQoL affects several components. Given the described effects on work and leisure time as well as on wages, the components *income and employment* and *leisure and recreation* could be supported by changing the overall economic structures. Similarly, the component *social relations and equality* could be supported, as participants would like to see a fairer distribution of income and wealth.

Almost one-third of the participants, especially farmers (10 participants), are of the perception that the added value of food should be stronger in order to support SQoL. They complained that food in Switzerland was too cheap and argued that, therefore, its prices should be increased. With higher prices, local small farms would also receive more income.

*“So that those who live here receive an added value from their product. That they not only produce more, but more or even less, but to a better added value.”* (IP 39, 30-59 years, male, forestry and agriculture, secondary school degree, rural peripheral municipality)

Regarding the concept of SQoL, this benefit would primarily affect the component *income and employment*. In addition, it could contribute to the component of *social relations and equality* because, according to the participants, smaller farms would be entitled to more income and the added value of products could contribute to a more equal distribution of income.

Almost a quarter of the participants sees sustainable tourism as beneficial to SQoL. It was mainly activities such as skiing, restaurant and hotel visits and farm holidays that were mentioned in this context. These participants argued that in their region, tourism was an important source of income that could reduce the migration of the local population. The participants feel that the type of tourism in highly frequented and well-known places is impossible and inappropriate for their region. In their perception, sustainable tourism could prevent negative effects on landscapes and the environment that commonly occur in places with strong conventional tourism.

*“We are not a tourist region like St. Moritz or other top destinations that only generate income with tourism. We are almost forced to practice sustainable, soft tourism, but we should try to get more out of it with little input, in a positive sense that it is really sustainable.”* (IP 31, 30-59 years, male, services, secondary school degree, rural central municipality)

Based on the participants’ statements, sustainable tourism would contribute to regional income and create jobs. With regard to the nine components of SQoL, it can thus contribute in particular to the component *income and employment*. In addition, sustainable tourism could be beneficial for the component *nature and landscape*, as the participants refer to moderate, ecological forms of tourism.

### Benefits to SQoL in the category of physical infrastructure, based on the perception of participants

In the category “physical infrastructure”, there are two types of benefits to SQoL: availability of alternatives to motorised private transport; and renewable energy (see Table 6). The majority of participants (79%) see alternatives to motorised private transport as beneficial to SQoL. They argued that motorised private transport caused carbon emissions, air pollution, hazards, noise and ground sealing and reduced the QoL. Despite this, most participants explained that they drove cars because public transport services were inadequate. Participants in the oldest age group (15 participants over 60 years) and in the youngest age group (31 participants between 16 and 29 years) would welcome closer access to public transport and more frequent public transport, as this would contribute to their freedom and thus to their QoL.

*“I would like to travel by train, but I have an orchestra rehearsal which finishes at half past ten in Zurich. I can get home in 25 minutes by car, by train I get home at twelve. Being home at half past ten is also quality of life. Then you can still have a drink, do something and go to sleep at twelve.”* (IP 73, 30–59 years, male, service, tertiary degree, rural central municipality)

**Table 6:** Benefits to SQoL in the category of physical infrastructure, based on the perception of participants, and the corresponding components from the SQoL concept

Benefits to SQoL in the category of physical infrastructure	Mentioned by …% of the 90 participants	Mentioned by … people out of the 90 participants	Concerns component … in the SQoL concept
Alternatives to motorised private transport are available.	78.8	71	Mobility Health and safety
Renewable energy for the home is available to everyone.	25.5	23	Income and employment Living Social relations and equality

With regard to the nine components of SQoL, alternatives to motorised private transport could, according to the descriptions of the participants, support the component *mobility*. This benefit to SQoL could also contribute to the component of *health and safety* since it is associated with the physical and mental health and safety of the population.

The second physical infrastructure, which almost a third of participants sees as favourable to SQoL, was renewable energy. Tenant interview participants complained that they could not freely choose their heating energy; they stated that they would like to see equal access to renewable energy for everybody. Homeowners, including farmers, said that they had installed solar panels or would like to do so because they saw solar panels as having financial and ecological advantages over other energy sources.

*“I have to say that we live really well here. What we still want is photovoltaics on our roof. We will probably change that.”* (IP 70, > 60 years, female, service, secondary school degree, rural central municipality)

Based on the participants’ statements, renewable energy could benefit the SQoL component *income and employment*, as some of the participants saw this as an option to save costs. In addition, the component *living* could also benefit, as the participants primarily discussed heating private living spaces. Some of the participants also opined that renewable energies should be equally available to everyone. In this sense, renewable energies could contribute to the component of *social relations and equality*.

## Discussion

This article aimed to evaluate the perception of Swiss rural inhabitants on beneficial factors for SQoL and to derive recommendations for local decision-makers. The rural participants concretely consider the following factors as beneficial to SQoL: access to renewable energy, alternatives to motorised private transport, seasonal and locally produced products, local services and leisure offers, common welfare economy, local products, sustainable tourism, changes in agricultural policies, nature and species protection, environmental habits, sufficiency, civil engagement, sustainability and environmental education for specific target groups, laws, sanctions and price regulations and policy focused on SQoL.

These results indicate that a relatively large proportion of the rural inhabitants had rather concrete ideas and awareness of ways how SQoL could be supported. Three-quarters of all interview participants named at least one benefit for SQoL that concerned attitudes and values, social and legal norms, economic structures or infrastructure.

The benefits for SQoL in all four categories affect several or all of the SQoL concept’s components (see Tables 3, 4, 5, 6 above). In addition, all nine components are targeted by the benefits for SQoL discussed in the interviews. The rural inhabitants in the interviews seem not to see the benefits as unilateral or limited to one component. It can be interpreted, that measures to support SQoL should simultaneously address attitudes and values, social and legal norms as well as physical infrastructure and economic structures. Besides direct official or state intervention, the mentioned benefits concern individuals and their life circumstances. Current discourses on sustainable development in the literature have a strong claim on complementary and comprehensive transformations. Accordingly, innovations should ideally address all three dimensions of sustainability (social, economy, ecology) at the same time [47]. With the inclusion of the social and economic dimensions into measurements, (sustainable)QoL would be addressed more strongly.

The category of physical infrastructure includes mobility and renewable energies, two technically complex areas that represent key challenges in rural regions of Switzerland [48, 49]. In addition, both areas primarily relate to the environmental dimension of sustainable development. A relatively large proportion of the rural inhabitants considered it important to create an environment that would facilitate more ecological behaviour. Infrastructures, such as renewable energy and sustainable mobility, contribute to the attractiveness of rural areas and, thus, to QoL and enable ecological behaviours [48–52]. Focus on changing structures, rather than individual attitudes and behaviour is also demanded in the literature on social and sustainable transitions [53], whereby the importance of the long-term policy, the election and decisions for adequate infrastructure is emphasized [54]. Certain infrastructures are especially important for specific social groups. For example, public transport is in the rural context particularly relevant for the young and elderly age groups who have no driver’s licences as it allows access to education, work, medical care and social inclusion [55–57].

The benefits for SQoL placed in the category “economic structures” shows that a relatively large proportion of the rural inhabitants interviewed consider structural economic changes to be essential for SQoL. This echoes, for example, Höflehner and Meyer [17] who recommend the promotion of regional value chains using decentralised technologies. The public perception of high-quality regional and seasonal food, farm shops and weekly markets can lead to social trends and shifts in values [17]. The resultant increase in added value created through local products provides local actors with economic opportunities that are both, realistic and sustainable [17, 58, 59]. Appreciation of the local services, the community, culture and identity associated with these products can contribute to social and cultural innovations and charitable activities [17]. The associated values are often independent of material claims [17] and contribute highly to the QoL of individuals. The enhancement of local productions, services and culture is especially important in light of local employment. The majority of the population in rural areas has a secondary degree and works in the field of agriculture, services and production. An existing functioning local economy could generate employers and, thus, more employment for the rural inhabitants.

The results in the category “attitudes and values” represent an important starting point for supporting opportunities in other categories. It would be difficult for changes in the environment, including physical infrastructure and economic structures, to arise independently of attitudes and values, social and legal norms or the transformation in society’s attitude [60]. In a democracy, decisions to support SQoL are mainly made by the part of society eligible to vote. The mindset of society and politics steers a paradigm shift towards sustainable development [60].

Our qualitative study and its derived recommendations must be also considered in light of its limitations. Depending on the context (e.g. the infrastructure), its degree of periphery and national legislation, the rural residents' may weigh the benefits for SQoL differently or even consider other aspects as benefits. In particular, the detailed measures to enhance SQoL should be determined in light of the specific contexts. Future studies could evaluate the perception of rural residents during the development of such measurements for SQoL. Knowing the perceptions might help to evaluate if the measurements are adequate to the context and if inhabitants can benefit from them. In addition, future studies could reflect the perceptions of urban residents to find relevant factors to enhance SQoL in urban areas or compare the perceptions of rural and urban inhabitants. The latter might contribute to the understanding of the mutual effects of rural and urban areas on SQoL.

## Conclusion

In conclusion, according to the interviewed rural inhabitants, challenges and potentials for SQoL mainly affect key areas of society that are mutually reinforcing in the investigated rural Swiss regions. Attitudes and values guide social and legal norms, and social and legal norms can guide both—physical infrastructure and economic structures.

The following recommendations for decision-makers can be derived from the results of this research article: (1.) The support of applied environmental and sustainability education in schools and the extracurricular context in a targeted group-specific manner seems crucial. For example, tips on more specific resource-efficient behaviour and a deeper understanding of SQoL could be given to contribute to behaviours and political voting in a way that is in the interest of SQoL. Likewise, the participatory involvement of the population could be facilitated and bottom-up processes thus initiated; these are important for regional sustainable development [61]. With regard to the ecological dimension of sustainable development, it can be recommended (2.) to make renewable energies available for everyone, including tenants, and to create incentives for the use of sustainable mobility. For cultural and social life and the fair distribution of income, it is recommended (3.) to promote the local productions and services and thus cyclic economy and ecology [17], and (4.) to involve economic actors in supporting SQoL through appropriate legal conditions, regulations and incentives.

## Data Availability

The full dataset generated and analysed during the current study is not publicly available as the manuscripts are Swiss German and very extensive. Insights into the used data are given in the results chapter in the form of quotations. However, the full dataset is available from the corresponding author on request.

## Appendix

See Table 7.

**Table 7:** Sociodemographic characteristics of the participants

Feature	Characteristics	Interview participants
Sex	Male	44
	Female	46
Age	16–29 years	26
	30–59 years	38
	60 < years	26
Highest education	Basic school degree	11
	Secondary school degree	61
	Tertiary degree	18
Employment sector	Forestry/agriculture	13
	Trade/industry	9
	Services	36
	Unemployed	1
	In education	15
	Retired	16
Municipality type (derived from the categorization by the Swiss Federal Office for the Environment, FOEN, 2012)	Peri-urban municipality	47
	Rural central municipality	28
	Rural peripheral municipality	15

*n* = *90*

## Footnotes

1 We define the term "quality of life" here as it is understood in several disciplines, such as psychology, philosophy and economics. It is mostly used to describe or measure the fulfilment of human needs and the satisfaction of individuals and groups with various aspects of life and is, thus, defined as a multidimensional construct [1, 2]. In many cases, so-called subjective and objective indicators are used for the measurement. For subjective indicators, individuals are asked about their assessment of their life satisfaction with various areas of life. Objective indicators include numbers, for example, about income or life expectancy and are often used to collect standardized data as they are seen as more valid for social comparisons [1–3]. However, in this article, we conducted interviews about possible improvements in the linkage of quality of life and sustainability and did, thus, not measure the quality of life of the individuals.

## Code availability

Codebook and questionnaire are available in the supplementary material Wiesli et al. [21].
